# Early Prediction of Diabetic Macular Edema via Machine Learning Survival Analysis on Checkup Data

**DOI:** 10.1016/j.xops.2026.101262

**Published:** 2026-06-01

**Authors:** Yusuke Kashiwagi, Katsuyuki Chida, Ayaka Hananoe, Ryoichi Ishibashi, Masaya Koshizaka, Yoshiro Maezawa, Yosuke Inaba, Yoko Takatsuna, Tomoaki Tatsumi, Hiroko Inoue, Hanae Wakabayashi, Tetsuo Ishikawa, Akiko Hanai, Koutaro Yokote, Katsuhiko Asanuma, Eiryo Kawakami

**Affiliations:** 1 Department of Artificial Intelligence Medicine, Graduate School of Medicine, Chiba University, Chiba, Japan; 2 Department of Nephrology, Graduate School of Medicine, Chiba University, Chiba, Japan; 3 Predictive Medicine Special Project (PMSP), RIKEN Center for Integrative Medical Sciences (IMS), RIKEN, Kanagawa, Japan; 4 Division of Applied Mathematical Science, RIKEN Center for Interdisciplinary Theoretical and Mathematical Sciences (iTHEMS), RIKEN, Kanagawa, Japan; 5 Division of Diabetes, Endocrinology and Metabolism, Department of Medicine, Kimitsu Chuo Hospital, Kisarazu, Japan; 6 Department of Endocrinology, Hematology, and Gerontology, Graduate School of Medicine, Chiba University, Chiba, Japan; 7 Center for Preventive Medical Sciences, Chiba University, Chiba, Japan; 8 Clinical Research Center, Chiba University Hospital, Chiba, Japan; 9 Department of Ophthalmology, Chiba Rosai Hospital, Ichihara, Japan; 10 Department of Ophthalmology and Vision Science, Graduate School of Medicine, Chiba University, Chiba, Japan; 11 Department of Extended Intelligence for Medicine, The Ishii-Ishibashi Laboratory, Keio University School of Medicine, Tokyo, Japan; 12 Collective Intelligence Research Laboratory, Graduate School of Arts and Sciences, The University of Tokyo, Tokyo, Japan; 13 Faculty of Informatics, Chiba University, Chiba, Japan

**Keywords:** Claims data, Diabetic macular edema, Health checkups, Machine learning, Survival analysis

## Abstract

**Purpose:**

To predict the risk of diabetic macular edema (DME) onset and to identify features of the risk subgroups.

**Design:**

Population-based observational study with a case-control design.

**Subjects and Controls:**

Health checkup and diagnosis data from the JMDC claims database (January 2005–July 2020), one of the largest Japanese epidemiological databases, were used. From 272 337 individuals diagnosed with type 2 diabetes (International Classification of Diseases, 10th Revision E11), we analyzed 2368 pairs of DME and non-DME individuals, which matched 1:1 by the balancing score calculated from regression analysis of DME with sex, age, months of observation, number of checkups, duration of diabetes, and months until the first checkup.

**Methods:**

We employed a multivariate Cox proportional hazards model, regularized Cox models, and a random survival forest (RSF). These models were trained via ID-level bootstrap resampling using 43 health checkup variables (missing ratio <50%) and 404 high-incidence diseases within 6 months, along with age, sex, and duration of diabetes mellitus, to assess DME risk. Temporal changes in RSF-predicted risk scores were analyzed using nonlinear modeling techniques.

**Main Outcome Measures:**

Concordance index (C-index), integrated Brier score (IBS), and cumulative/dynamic mean area under the receiver operating characteristic curve (AUC).

**Results:**

Thirteen checkup items and 44 disease history variables were significantly associated with the onset of DME. The RSF identified 43.8% of DME cases >5 years prior to onset, with a specificity of 85.5%. The RSF achieved median C-index, IBS, and mean AUC values of 0.694 (95% confidence interval, 0.688–0.697), 0.181 (0.179–0.184), and 0.750 (0.739–0.756), respectively, outperforming both multivariate and univariate Cox models. Three distinct DME risk subgroups were suggested by temporal changes in the RSF-predicted risk score. Predictors of DME onset varied markedly among these subgroups. In the explicit high-risk subgroup, urinary protein and urinary sugar were highly important, and liver function-related blood tests, such as alanine transaminase and γ-gultamyltransferase, were also ranked high in variable importance metrics. Anemia-related laboratory tests were associated with DME development only in this subgroup.

**Conclusions:**

Random survival forest demonstrated superior performance in relative risk prediction of DME using health checkup data. External validation remains an essential prerequisite before any clinical application.

**Financial Disclosures:**

Proprietary or commercial disclosure may be found in the Footnotes and Disclosures at the end of this article.

The global rise in diabetes mellitus highlights diabetic retinopathy, a common diabetes mellitus complication, as a major health challenge.^1^^,^^2^ Diabetic macular edema (DME), a severe diabetic retinopathy manifestation affecting central vision, impacts ∼21 million people worldwide and is a leading cause of vision loss.^3^ Anti-VEGF intravitreal injections improve long-term visual outcomes but are costly and require repeated treatments.^4^^,^^5^ A series of ETDRS has shown that laser photocoagulation, the standard DME care until the advent of anti-VEGF therapy, is effective for patients with early DME.^6^ Recently, early anti-VEGF therapy has also been reported to preserve vision.^7^ Therefore, early diagnosis and treatment of DME are desirable to improve visual prognosis.

A variety of risk factors for DME are known and serve as indicators for early prediction of DME onset.^8^ Poor diabetes control, as indicated by elevated hemoglobin A1c (HbA1c) and fasting blood glucose (FBG), increases the risk of DME.^9^, ^10^, ^11^ Lifestyle factors such as hypertension, hyperlipidemia, and obesity also contribute to DME risk.^10^, ^11^, ^12^ Diabetic kidney disease (DKD), another microvascular complication, is closely linked to DME, with hypertension from renal impairment adding further risk.^13^ Imaging techniques, such as OCT and fundus imaging, have enabled early and accurate diagnosis of DME with high sensitivity and specificity.^14^, ^15^, ^16^, ^17^ Nonetheless, a general risk assessment tool is required for prescreening because these diagnostic models are based on patients who present at the hospital with symptoms, and OCT and fundus imaging are limited to certain facilities.

We focused on Japanese annual health checkups mandated by employers to develop a DME risk screening tool using only data readily available before disease onset. More than 60% of the population aged ≥20 years undergoes the checkups.^18^ The checkups include ∼30 items such as questionnaires, blood/urine tests, and x-rays.^19^ Notably, the checkup also includes many key items associated with the development of DME, such as HbA1c, FBG, urinary sugar, blood pressure, blood lipids, and body mass index. We hypothesized that these data, with diagnostic information from claims data, could enable the early assessment of DME risk. A prediction model in a binary classification framework is unsuitable for diseases with long-term progression, as DME typically progresses over 5 to 10 years. A survival analysis framework that considers right-censored samples as sensors is an appropriate choice for addressing this problem.^20^ Traditional methods, such as the Cox proportional hazards model,^21^ were developed for small data sets with limited explanatory variables. On the other hand, machine learning models, such as least absolute shrinkage and selection operator, random forests, and deep learning models, can handle high-dimensional, heterogeneous data,^22^ including health checkups and claims data. Thus, we used random survival forests (RSFs)^23^ to predict DME onset through checkup and claims data.

This study aimed to assess each diabetic patient’s relative risk of DME onset based on annual checkup data and to determine how the risk changed prior to DME onset. We also revealed the heterogeneous impact of risk factors on DME development through machine learning–based survival analysis.

## Methods

### Compliance with Ethical Guidelines

This study was approved by the Institutional Review Board of Chiba University School of Medicine (approval number: 4175) under the “Ethical Guidelines for Medical and Health Research Involving Human Subjects” of the Ministry of Health, Labour and Welfare. Informed consent was waived due to the use of anonymized, deidentified data. This study is registered with the UMIN Clinical Trial Registry (registry number: UMIN000042242). This study was conducted in accordance with the principles of the Declaration of Helsinki.

### Data Source and Study Population

This retrospective case-control study used the JMDC claims database, one of the largest Japanese epidemiological databases, based on Japan’s universal health insurance system.^24^ It includes drug prescription, International Classification of Diseases, 10th Revision–coded disease history, and health checkup data from 216 societies and approximately 6.1 million insured persons as of July 2020.^25^

**Figure 1:**
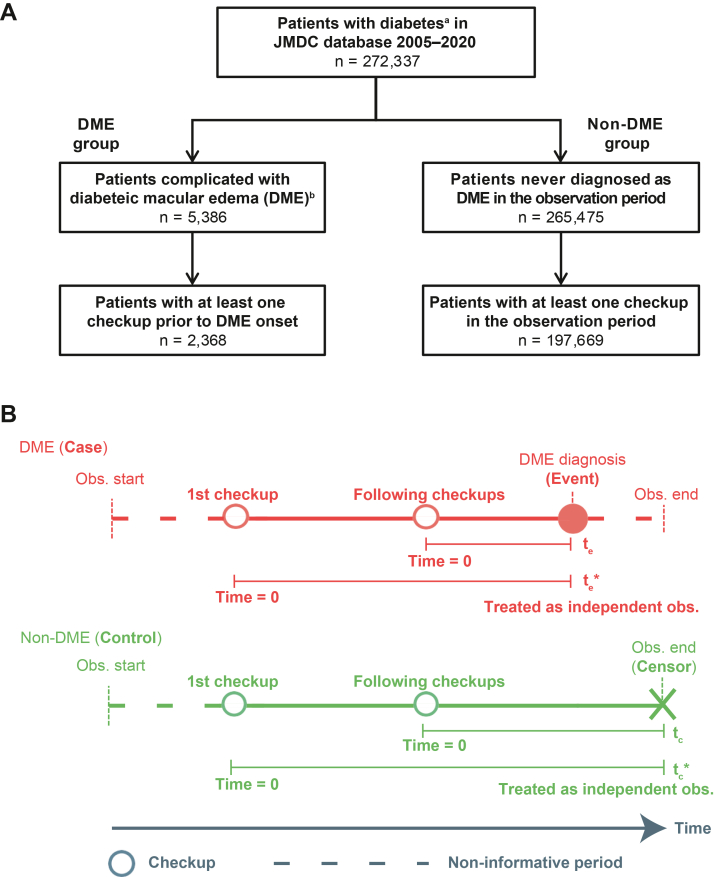
Selection of DME and non-DME groups and study timeline. (**A**) ^a^Patients with diabetes were defined as those with the ICD-10 code E11 during the observation period. ^b^Individuals in the DME group were defined as those with ≥1 diagnosis of “type 2 diabetic macular edema,” “diabetic macular edema,” “cystoid macular edema,” or “diabetic maculopathy” without a “suspected” disease flag. (**B**) Each health checkup record from a patient was treated as an independent observation in the survival analysis, with t_e_ representing the event time, t_c_ the censoring time, and Obs. the observation. DME = diabetic macular edema; ICD-10 = International Classification of Diseases, 10th Revision.

We extracted data for 272 337 individuals from the JMDC database (January 2005–July 2020) who were diagnosed with type 2 diabetes (International Classification of Diseases, 10th Revision: E11). From these, 5386 patients with “type 2 diabetic macular edema,” “diabetic macular edema,” “cystoid macular edema,” or “diabetic maculopathy” formed the DME group. Diagnoses flagged as “suspected” were not included. A suspected flag denotes a provisional diagnosis pending further examination and is removed upon confirmation of the disease by subsequent examination. For analysis, 2368 DME patients with ≥1 health checkup before onset were included. Type 2 diabetic patients without DME but with ≥1 health checkup were selected as controls (n = 197 669) (Fig. 1A). To reduce computational cost and mitigate sample imbalance, we implemented a matched sampling strategy. We matched controls 1:1 to the DME patients using a balancing score calculated from regression analysis of DME with sex, age, months of observation, number of checkups, duration of diabetes, and months until the first checkup, using MatchIT R package^26^ in R, which yielded 2368 matched pairs. As a sensitivity analysis to assess the effect of prevalence distortion introduced by matching, we evaluated RSF performance on test sets constructed from patients with diabetes who were not used for model training, assuming a DME prevalence of 2%. In an additional sensitivity analysis, matching was performed using only sex and age.

### Definition of Health Checkup Items

The JMDC data include 54 items, such as physical measurements, blood pressure, laboratory tests, symptoms, medical history, lifestyle interview, and health guidance. Of these, 43 items with a missing ratio of <50% were used, along with age, sex, and duration of DM, defined as the time from the first diagnosis of diabetes to the health checkup. Additionally, 404 high-incidence diseases (with a prevalence >1% among all patients) within a 6-month period preceding the checkups were included as predictors. For chronic diseases defined operationally for this study, histories prior to the checkup were used regardless of the 6-month limit. This approach was chosen to reflect the long-term nature of chronic diseases and their relevance to current health status. Diseases were encoded as binary indicators, with the presence of any diagnosis coded as 1 regardless of the number of occurrences.

For survival analyses, we implemented a parameter-specific sample-and-hold approach,^27^^,^^28^ which adopted the last recorded measurement values, to impute missing values. Missing values before the first measurement were imputed using the median (continuous) or mode (categorical). In a sensitivity analysis, multivariate imputation by chained equations was used to impute missing values. Categorical variables, all binary or ordinal, were converted to numeric values. These variables were used as raw values in the RSF and as standardized values in the multivariate Cox models.

### Machine Learning–Based Survival Analysis

A multivariate Cox proportional hazards model and regularized ones, Cox least absolute shrinkage and selection operator, Cox Ridge, and CoxNet,^29^ were used for survival analysis under the proportional hazard assumption using the scikit-survival Python package.^30^ The regularization strength (α) was optimized via grid search using the Concordance index (C-index) as the metric.

Random survival forest is a nonlinear survival analysis method based on random forests. Each patient’s hazard function is estimated by averaging hazard functions from all trees. The RSF model, implemented in the scikit-survival Python package,^30^ was used with default settings, except for n_estimators = 200, min_samples_split = 10, and min_samples_leaf = 15.

Fig. 1B illustrates the definition of survival time, event occurrence, and censoring used in the survival analysis.

### Evaluation of Model Performance

The performance of the survival model was evaluated using 3 metrics below. Confidence intervals (CIs) were estimated using bootstrap resampling with replacement (200 iterations) and the percentile method.

#### C-index

The C-index is defined as the proportion of predicted risk scores that correctly rank the timing of events in comparable pairs.

#### Integrated Brier score

The time-dependent Brier score is similar to mean-squared error and considers the error between predicted probabilities and actual events. The integrated Brier score (IBS) is the integral of the time-dependent Brier score weighted by the time to event over the given time interval. In this study, we used a time interval from 12 to 120 months before the event.

#### Mean cumulative/dynamic area under the receiver operating characteristic curve

This is an extension of area under the receiver operating characteristic curve (AUC), defined as a time-dependent measure of sensitivity and specificity. The mean AUC is the integral of the cumulative/dynamic AUC weighted by the Kaplan-Meier estimator over the given time interval. We used the same time interval as in IBS.

### Statistical Analysis

Participant characteristics before and after matching were presented as numbers (%) for categorical variables and means (standard deviation) for continuous variables and tested using the Wilcoxon rank sum test and the Pearson χ^2^ test.

Mixed-effects Cox models in the coxme R package were used to analyze the association between DME onset and health checkup data and disease history. The models included health checkup items and disease history as independent variables, with patient ID as a random effect to account for repeated measures. Age at the health checkup was controlled for to adjust for its natural increase.

We applied the Self-Starting Nls Logistic Model (SSlogis), a type of logistic function, to examine temporal changes in the risk scores of developing DME because the risk of developing DME is essentially increasing. The equation used is:

RS=Asym/(1+exp((xmid–t)/scal))

where *RS* is the mean of the RSF-predicted risk score for DME derived from 10-fold cross-validation and *t* is the time to outcome. The SSlogis model has 3 parameters: *Asym* represents the asymptotic value as time approaches infinity, *xmid* is the time at which SSlogis reaches half of *Asym*, and *scal* is the reciprocal of the slope of SSlogis, where a smaller *scal* indicates a faster increase. Random effects for these parameters were incorporated using the nlmer R function. Ward’s hierarchical clustering was performed with the hclust R function, using Euclidean distances based on the random effects. Cluster label stability was evaluated using the adjusted mutual information score between the original data set and bootstrap-resampled training data sets, with the test data set held fixed.

All statistical analysis was performed using R (R Project for Statistical Computing), version 4.4.1. Statistical significance was determined at P or adjusted *P* < 0.05.

### Data Resource and Availability

The data used in this study are unavailable publicly due to a license agreement with JMDC Inc, but access may be granted through collaborative research agreements with the corresponding authors. The analysis source code is available on GitHub (https://github.com/eiryo-kawakami/JMDC_DME_paper).

## Results

### Characteristics of the Study Population

Diabetic patients in the DME group had different characteristics compared with those in the non-DME group (Table S1, available at www.ophthalmologyscience.org). The DME group had a higher proportion of men, a longer observation period, and nearly 2 years longer diabetes duration. It was younger at the onset of diabetes. Probably due to the longer observation period, the DME group had more health checkups during the observation period. In contrast, there were no significant differences in age at the start of observation and time until the first health checkup. Matching yielded 2368 matched pairs of DME and non-DME, with no significant differences observed for any variables.

### Associations Between Variables and DME Onset

Mixed-effects Cox models were used to analyze the statistical association between checkup items (Table S2, available at www.ophthalmologyscience.org) and disease history (Table S3, available at www.ophthalmologyscience.org) and the onset of DME, adjusting for age. Of the 44 items and 404 diseases, 13 items and 44 diseases showed significant associations with DME onset:

#### Physical measurements

Body mass index and waist circumference were positively associated.

#### Blood and urine tests

Hemoglobin A1c and urinary sugar and protein levels were positively associated.

#### Food and lifestyle questionnaires

Frequency of alcohol consumption, walking speed compared with peers, and willingness to receive lifestyle guidance were inversely associated, while walking ≥1 hour per day was positively associated.

#### Disease history and medication questionnaires

A history of stroke or renal failure and the use of antihypertensive drugs, insulin, or hypoglycemic drugs were positively associated.

#### Disease history from diagnoses

Among 404 diseases, 44 showed significant positive associations with DME. The most frequent International Classification of Diseases, 10th Revision blocks were eye diseases (H00–H59, 14), endocrine/metabolic disorders (E00–E90, 12), circulatory (I00–I99, 4), and respiratory (J00–J99, 4). Endocrine/metabolic disorders included diabetes complications such as DKD, proliferative diabetic retinopathy, and diabetic peripheral neuropathy (DPN) and metabolic abnormalities like dyslipidemia and hyperuricemia.

### DME Onset Prediction with Machine Learning Survival Analysis

Table S4 (available at www.ophthalmologyscience.org) summarizes DME onset predictions using RSF and Cox models. The RSF demonstrated a C-index of 0.694 (95% CI, 0.688–0.697), IBS of 0.181 (95% CI, 0.179–0.184), and a mean AUC of 0.750 (95% CI, 0.739–0.756), outperforming multivariate and univariate Cox models across all metrics. Even when compared with the best-performing comparator model, the RSF model showed a higher C-index, with a difference of 0.015 (95% CI, 0.005–0.022). Diseases, like chronic gastritis, DKD, and fatty liver, contributed minimally to the prediction, with C-index values near 0.5 from the univariate Cox model. The multivariate Cox model, incorporating all variables, improved performance to a C-index >0.665, whereas regularization yielded only a marginal improvement.

**Figure 2:**
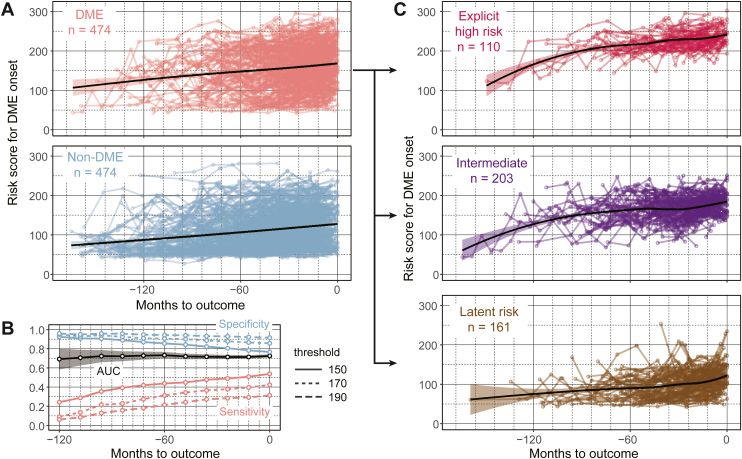
Temporal changes in DME incidence risks predicted by RSF. (**A**) The RSF risk score for DME and non-DME groups plotted against time to outcome (onset for the DME group, censored for the non-DME group). Fitted curves using the locally estimated scatterplot smoothing method are shown as solid lines, with the standard error shaded. (**B**) Area under the receiver operating characteristic curve, sensitivity, and specificity of predicting DME onset based on the RSF risk score, with different line types representing thresholds of 150, 170, and 190. (**C**) Temporal changes in the RSF risk score across 3 subgroups as stratified by the nonlinear mixed-effects model. AUC = area under the receiver operating characteristic curve; DME = diabetic macular edema; RSF = random survival forest.

Random survival forest-predicted risk scores were increasing by ∼5 points per year as DME onset approached (Fig. 2A). The DME group had an average risk score of 50 points higher than the non-DME group. The AUC for RSF-predicted risk remained ∼0.72 over time (Fig. 2B). The relationship between thresholds and performance metrics at 5 years prior to DME diagnosis was examined as a reference to facilitate interpretation of the results. Risk score threshold of 150 points yielded sensitivity of 43.8% and specificity of 85.5%. At thresholds of 170 points and 190 points, sensitivity dropped (31.3% and 21.4%, respectively), but specificity rose (90.5% and 94.2%). We then stratified changes in RSF-predicted risk score over time, employing a nonlinear mixed-effects model, and identified 3 distinct patterns (Fig. 2C): (1) a rapid rise about 10 years before onset with sustained high risk (explicit high-risk subgroup), (2) gradual increase from moderate risk (intermediate-risk subgroup), and (3) persistently low risk until shortly before onset (latent-risk subgroup). The adjusted mutual information score was 0.613 (95% CI, 0.477–0.733), indicating moderate cluster stability.

### Factors Contributing to DME Onset Prediction

**Figure 3:**
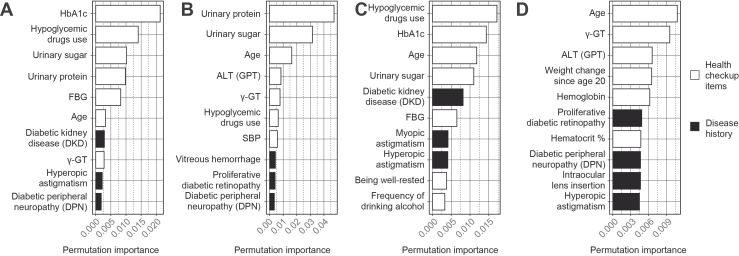
Evaluation of factors contributing to the development of DME using permutation importance. (**A**) Importance in all test data. (**B**) Importance in the explicit high-risk group. (**C**) Importance in the intermediate-risk group. (**D**) Importance in the latent-risk group. ALT = alanine transaminase; DME = diabetic macular edema; FBG = fasting blood glucose; GPT = glutamate pyruvate transaminase; HbA1c = hemoglobin A1c; SBP = systolic blood pressure; γ-GT = γ-gultamyltransferase.

Factor contributions to DME onset prediction by RSF were evaluated using permutation importance. Hemoglobin A1c was the strongest predictor, with the use of hypoglycemic medications as the second strongest (Fig. 3A). Urinary glucose and protein, key indicators of diabetes-related kidney damage, along with a history of DKD and DPN, emerged as top predictors of DME development. Fasting blood glucose, age, and γ-gultamyltransferase, which were all statistically significantly associated with DME development, were also important predictors. An examination of important factors for each of the 3 DME subgroups revealed that predictors varied considerably among the subgroups (Fig. 3B–D). In the explicit high-risk group, urine protein and urine sugar were highly important, and liver function-related blood tests such as alanine transaminase and γ-gultamyltransferase were also ranked highly (Fig. 3B). In the intermediate-risk group, DKD was characteristically important, and ocular conditions, including myopic astigmatism and hyperopic astigmatism, emerged as significant predictors, whereas urinary protein played a less important role (Fig. 3C). The latent-risk group was characterized by the high importance of weight change since age 20 (Fig. 3D). Anemia-related laboratory tests were associated with DME development only in this subgroup, such as blood hemoglobin concentration and hematocrit. Age remained an important predictor across all subgroups.

### Temporal Changes in Key DME Predictors

**Figure 4:**
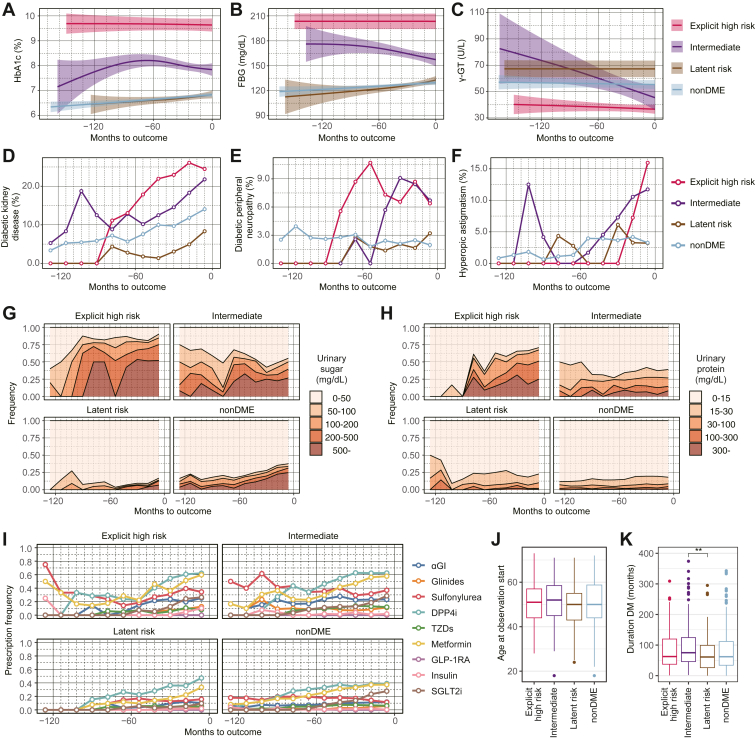
Visualization of important predictors of DME onset over time. (**A**–**C**) Fitted curves using the locally estimated scatterplot smoothing method are shown as solid lines, with the standard error shaded, for blood tests like HbA1c, FBG, and γ-GT. (**D–F**) For conditions like diabetic kidney disease, diabetic peripheral neuropathy, and hyperopic astigmatism, the frequency of occurrence was tabulated and plotted every 6 months. (**G**, **H**) For urinary sugar and protein, the percentages for each discrete level were tabulated every 6 months and presented in a stacked line graph. (**I**) For hypoglycemic drug prescriptions, prescription frequency for 9 major drug categories was derived from insurance claims data and plotted every 6 months. (**J**, **K**) Age at the beginning of the observation and duration of diabetes at the end of the observation are shown as boxplots. Differences between groups were determined using Welch *t* test across all pairwise combinations, and *P* values were adjusted using the Holm method. Significant differences are denoted with asterisks as follows: ∗*P* < 0.05, ∗∗*P* < 0.01. DM = diabetes mellitus; DME = diabetic macular edema; FBG = fasting blood glucose; HbA1c = hemoglobin A1c; γ-GT = γ-gultamyltransferase.

Finally, we examined temporal changes in key DME predictors across subgroups. Hemoglobin A1c and FBG were highest in the high-risk subgroup, moderate in the intermediate-risk subgroup, and comparable between the latent-risk and non-DME subgroup (Fig. 4A, B). γ-gultamyltransferase was consistently low in the explicit high-risk subgroup but decreased markedly in the intermediate-risk subgroup from a relatively high baseline (Fig. 4C).

More than 10% of the intermediate-risk subgroup had DKD consistently for >5 years before DME onset, while the explicit high-risk subgroup showed a sharp increase in DKD incidence starting ∼7 years before onset (Fig. 4D). Diabetic peripheral neuropathy incidence remained stable (∼2.5%) in the non-DME subgroup but increased 5 to 7 years before onset in DME subgroups, earliest in the explicit high-risk and latest in the intermediate-risk (Fig. 4E). Hyperopic astigmatism incidence rose 2 to 5 years before onset in all DME subgroups, with the sharpest rise in the explicit high-risk group (Fig. 4F). Severe proteinuria and glucosuria were evident in the explicit high-risk subgroup >5 years before onset (Fig. 4G, H). The intermediate-risk subgroup showed moderately elevated levels, whereas the latent-risk subgroup had low urinary sugar and protein comparable to non-DME. Despite persistent hyperglycemia, the explicit high-risk group received fewer hypoglycemic drug prescriptions than the intermediate-risk group (Fig. 4I). There was no significant difference in the age at the start of observation among subgroups (Fig. 4J). Diabetes duration at the end of observation was longest in the intermediate-risk group and shortest in the latent-risk group, with a significant difference (Fig. 4J, K).

### Sensitivity Analysis

Because the case-control design distorts the true prevalence of DME and complicates interpretation, we conducted the first sensitivity analysis in which the trained RSF model was kept fixed and evaluated on test data sets generated by random sampling from all non-DME individuals with DME individuals in the original test data while preserving an assumed DME prevalence of 2%. The performance on the new test data sets showed median C-index, IBS, and mean AUC values of 0.790 (95% CI, 0.765–0.815), 0.058 (95% CI, 0.055–0.061), and 0.832 (95% CI, 0.809–0.857), respectively.

Matching using variables that are not available at prediction time may amplify bias. In the second sensitivity analysis using a sex-matched and age-matched data set, the RSF achieved median C-index, IBS, and mean AUC values of 0.714 (95% CI, 0.713–0.716), 0.177 (95% CI, 0.174–0.179), and 0.755 (95% CI, 0.753–0.758), respectively.

The parameter-specific sample-and-hold approach for imputing missing values was not feasible when variables were retained over longer time periods; therefore, we used multivariate imputation by chained equations as the third sensitivity analysis. The RSF model with multivariate imputation by chained equations achieved median C-index, IBS, and mean AUC values comparable to those observed in the original analysis (Table S5, available at www.ophthalmologyscience.org).

## Discussion

This is the first study to demonstrate the utility of machine learning survival models for predicting DME onset, using only widely available data, such as health checkups and medical history. The RSF model could detect 43.8% of DME cases >5 years before onset, with a specificity of 85.5%. Furthermore, temporal changes in RSF-predicted risk scores suggested 3 distinct DME risk groups. The key predictors of DME development differed markedly among these risk groups. Our model is easily applicable and has potential for early DME detection. Further clinical research on prospective screening and early intervention based on these findings is encouraged to improve DME prognosis. The 2 sensitivity analyses—using the original prevalence and the sex-matched and age-matched dataset—demonstrated improved performance. The less restrictive matching strategy may have resulted in more clearly separable groups, yielding performance closer to what would be expected in clinical practice.

Gastrointestinal, cardiovascular, and respiratory diseases were associated with DME onset in statistical survival analysis. They were not previously identified as definitive risk factors for DME. Gastrointestinal conditions like chronic gastritis and reflux esophagitis may be associated with side effects of diabetes medications (e.g., metformin, glucagon-like peptide-1 receptor agonists), causing digestive issues.^31^^,^^32^ Cardiovascular diseases, more common in DME patients, may share microvascular damage as a common underlying factor with DME.^33^ Upper respiratory inflammation, as suggested by chronic sinusitis, allergic rhinitis, and bronchial asthma, may be associated with DME. Elevated inflammatory cytokines (e.g., interleukin-6, interleukin-8, monocyte chemoattractant protein-1) in chronic inflammation may increase vascular permeability and immune activation, contributing to DME development.^34^ Nonetheless, these diseases and statistically significant factors in RSF do not always improve prediction.^35^

Compared to univariate Cox regression, multivariate Cox regression substantially improved DME onset prediction, suggesting synergistic effects of multiple factors. Indeed, DME involves complex processes, including angiogenesis and inflammation, which interact during progression.^36^^,^^37^ Random survival forest further outperformed Cox models by leveraging population heterogeneity and diverse predictors across decision trees, whereas Cox models assume consistent factor contributions.

Although the subgroup stratification was exploratory and demonstrated only moderate stability, the 3 DME risk subgroups may reflect distinct underlying mechanisms. The explicit high-risk subgroup showed persistently high HbA1c and FBG, abrupt DKD and DPN onset about 7 years before DME, and low hypoglycemic agent use, suggesting poor hospital attendance and persistent hyperglycemia as a direct cause. The intermediate-risk subgroup showed prolonged diabetes with DKD, frequent hypoglycemic agent use, and gradual improvement in glycemic markers, suggesting renal impairment as a driver of DME. The latent-risk subgroup maintained good glycemic control and similar complication rates to non-DME, yet its DME onset remains unclear; RSF scores remained lower until onset, and factors such as capillary fragility might contribute, but we were unable to determine whether unobserved acute events influenced the outcomes or whether the RSF model failed to learn features associated with DME.

### Strengths and Limitations

The RSF model predicts DME up to 5 years before onset, using data only from annual health checkups and medical history. Specificity is critical for practical use because DME affects only 2% to 3% of diabetic workers aged <65 years. Nonetheless, this screening will be useful for planning examination frequency because fundus examinations are recommended for diabetic patients.^38^

This study has several limitations. First, the database includes only workers aged <65 years and their dependents. Future analyses should incorporate data from individuals aged ≥70 years. A second limitation is that insurance claims data often include provisional disease names and have limited sensitivity for certain diagnoses, which may compromise the accuracy of DME and other disease diagnoses. While removal of the “suspected” disease flag was intended to exclude patients ultimately judged not to have the disease after examination, this process is expected to reduce false-positive DME cases.^39^ However, some recorded disease codes may still correspond to provisional diagnoses assigned during the examination process. Third, the annual health checkup schedule provides limited temporal resolution, which may lead to missed acute diagnoses or transient clinical information. Fourth, individuals who attend health checkups regularly may have higher health awareness and better overall health status than the general population. This may introduce selection bias because our analyses were based on health checkup data. Finally, the JMDC database lacks clinical details, such as disease severity and outcomes. Validation using detailed clinical data from medical institutions is essential.

## Declaration of Generative AI and AI-Assisted Technologies in the Writing Process

During the preparation of this work the authors used Microsoft Copilot in order to assist with grammar checking and proofreading. After using this tool, the authors reviewed and edited the content as needed and take full responsibility for the content of the publication.
