# Supplementary material for: Early Prediction of Diabetic Macular Edema via Machine Learning Survival Analysis on Checkup Data
Source: Ophthalmol Sci. 2026 Jun 1;6(8):101262. doi: 10.1016/j.xops.2026.101262 (PMC13355755; doi:10.1016/j.xops.2026.101262)
Supplement: Table S1 [file mmc1.pdf]

Table S1 Characteristics of DME and non-DME populations before and after the matching

	Pre-match characteristics			Post-match characteristics		
	Non-DME (n=197,669)	DME (n=2,368)	P value	Non-DME (n=2,368)	DME (n=2,368)	P value
**Gender**			**< .001**			.185
Female	49,890 (25.2%)	443 (18.7%)		408 (17.2%)	443 (18.7%)	
Male	147,779 (74.8%)	1,925 (81.3%)		1,960 (82.8%)	1,925 (81.3%)	
Age at start of observation	50.4 (10.0)	50.6 (9.32)	.997	50.3 (9.87)	50.6 (9.32)	.535
Duration of observation	64.4 (39.9)	77.0 (42.1)	**< .001**	77.3 (43.8)	77.0 (42.1)	.927
Duration of diabetes at end of observation	56.2 (49.2)	78.8 (60.8)	**< .001**	77.0 (61.0)	78.8 (60.8)	.153
Months until the first health checkup from the start of observation	11.1 (16.0)	11.4 (16.4)	.709	10.8 (14.6)	11.4 (16.4)	.839
Number of health checkups	4.55 (3.25)	5.41 (3.63)	**< .001**	5.46 (3.53)	5.41 (3.63)	.489
**Survival analysis**						
Follow-up duration	43.1 (33.1)	34.5 (30.4)	**< .001**	49.4 (35.8)	34.5 (30.4)	**< .001**
Checkup frequency during the follow-up period	4.45 (3.25)	3.56 (3.04)	**< .001**	5.46 (3.53)	3.56 (3.04)	**< .001**

P values were calculated with Wilcoxon rank-sum tests for continuous variables and Pearson’s Chi-squared test for categorical variables. Statistically significant differences ($P < 0.05$) are indicated in bold. Numbers in parentheses indicate standard deviations (SD) for continuous variables and percentages for categorical variables.
