# Supplementary material for: Early Prediction of Diabetic Macular Edema via Machine Learning Survival Analysis on Checkup Data
Source: Ophthalmol Sci. 2026 Jun 1;6(8):101262. doi: 10.1016/j.xops.2026.101262 (PMC13355755; doi:10.1016/j.xops.2026.101262)
Supplement: Table S2 [file mmc2.pdf]

Table S2 Association of health checkup items with DME onset

	Non-DME<br>n = 2,368<br>12,927 tests	DME<br>n = 2,368<br>8,435 tests	Cox coefficient	Cox adjusted P value
Gender Female	1,704 (13.2%)	1,273 (15.1%)	-0.171	1.000
BMI (kg/m^2)	26.1 (4.72)	26.2 (4.50)	0.079	**<0.001**
Waist (cm)	90.1 (11.4)	90.6 (11.3)	0.027	**<0.001**
Any past medical history	6,717 (52.0%)	4,727 (56.0%)	0.170	0.130
Any subjective symptoms	5,230 (40.5%)	3,572 (42.3%)	0.132	0.327
Any objective symptoms	1,546 (12.0%)	994 (11.8%)	-0.177	1.000
Systolic blood pressure (mmHg)	128 (16.5)	132 (18.2)	0.003	1.000
Diastolic blood pressure (mmHg)	79.5 (11.3)	80.2 (11.9)	0.003	1.000
Neutral fat (mg/dL)	147 (116)	165 (151)	0.000	1.000
HDL cholesterol	55.4 (15.4)	54.4 (14.8)	-0.006	0.358
LDL cholesterol	122 (32.5)	122 (35.3)	-0.002	0.510
AST (GOT) (U/L)	26.5 (14.4)	25.6 (15.4)	-0.003	1.000
ALT (GPT) (U/L)	33.3 (24.3)	31.7 (24.5)	-0.003	0.058
γ-GT (GGT) (U/L)	54.7 (62.8)	52.3 (64.8)	-0.001	0.463
Fasting blood glucose (mg/dL)	125 (38.2)	160 (65.1)	0.000	1.000
HbA1c (%)	6.67 (1.28)	7.99 (2.01)	0.063	**<0.001**
Urinary sugar (mg/dL)			0.141	**<0.001**
0-50	9,725 (75.2%)	4,264 (50.6%)		
50-100	398 (3.1%)	383 (4.5%)		
100-200	550 (4.3%)	679 (8.0%)		

	Non-DME n = 2,368 12,927 tests	DME n = 2,368 8,435 tests	Cox coefficient	Cox adjusted P value
200-500	502 (3.9%)	753 (8.9%)		
500-	1,412 (10.9%)	1,951 (23.1%)		
Urinary protein (mg/dL)			0.199	**<0.001**
0-15	10,162 (78.6%)	5,197 (61.6%)		
15-30	1,274 (9.9%)	832 (9.9%)		
30-100	706 (5.5%)	844 (10.0%)		
100-300	316 (2.4%)	649 (7.7%)		
300-	145 (1.1%)	520 (6.2%)		
Hematocrit (%)	45.2 (3.99)	44.6 (4.33)	-0.018	1.000
Hemoglobin (g/dL)	15.0 (1.41)	15.0 (1.54)	-0.056	1.000
RBC count ($10^4/mm^3$)	490 (54.6)	489 (59.0)	0.002	0.097
Abnormal findings in electrocardiogram	2,241 (17.3%)	1,398 (16.6%)	0.166	0.473
Current smoking	3,886 (30.1%)	2,717 (32.2%)	-0.200	1.000
Eating speed compared with other people			0.034	1.000
Fast (=1)	3,847 (29.8%)	2,445 (29.0%)		
Normal (=2)	5,640 (43.6%)	3,548 (42.1%)		
Slow (=3)	870 (6.7%)	585 (6.9%)		
Eating dinner within 2 hours before bedtime at least 3 times a week	3,552 (27.5%)	2,457 (29.1%)	0.106	1.000

	Non-DME n = 2,368 12,927 tests	DME n = 2,368 8,435 tests	Cox coefficient	Cox adjusted P value
Snacking after dinner (night meal other than 3 meals) at least 3 times a week	1,167 (9.0%)	912 (10.8%)	-0.016	1.000
Skipping breakfast at least 3 times a week	1,615 (12.5%)	1,216 (14.4%)	0.034	1.000
Drinking alcohol			-0.379	**<0.001**
Every day (=3)	2,879 (22.3%)	1,462 (17.3%)		
Occasional (=2)	3,677 (28.4%)	2,181 (25.9%)		
Rarely (=1)	4,733 (36.6%)	3,399 (40.3%)		
Amount of alcohol consumed per day on drinking days			-0.027	1.000
Less than 1 cup of sake (=1)	3,592 (27.8%)	2,245 (26.6%)		
1 to 2 cups of sake (=2)	2,669 (20.6%)	1,339 (15.9%)		
2 to 3 cups of sake (=3)	1,240 (9.6%)	735 (8.7%)		
over 3 cups of sake (=4)	466 (3.6%)	289 (3.4%)		
Being well-rested	6,518 (50.4%)	3,646 (43.2%)	-0.129	0.615
Use of antihypertensive medications	4,632 (35.8%)	2,727 (32.3%)	0.404	**<0.001**
Use of insulin injections or hypoglycemic drugs	5,139 (39.8%)	4,892 (58.0%)	0.876	**<0.001**
Dyslipidemia medication use	3,485 (27.0%)	2,173 (25.8%)	0.169	0.349

	Non-DME<br>n = 2,368<br>12,927 tests	DME<br>n = 2,368<br>8,435 tests	Cox coefficient	Cox adjusted P value
History of stroke (cerebral hemorrhage, cerebral infarction, etc.)	210 (1.6%)	231 (2.7%)	0.698	**0.001**
History of heart disease (angina pectoris, myocardial infarction, etc.)	742 (5.7%)	443 (5.3%)	0.078	1.000
History of chronic renal failure	114 (0.9%)	98 (1.2%)	0.782	**0.036**
Anemia	12,734 (98.5%)	8,310 (98.5%)	-0.089	1.000
Has gained more than 10 kg from his/her weight at age 20	6,349 (49.1%)	3,411 (40.4%)	-0.166	0.222
At least 2 days a week of light, sweaty exercise for at least 30 minutes per session for at least 1 year	2,848 (22.0%)	1,702 (20.2%)	-0.090	1.000
At least 1 hour per day of walking or equivalent physical activity in daily life	3,559 (27.5%)	2,193 (26.0%)	0.196	**0.002**
Walking faster than their peers of approximately the same age	4,193 (32.4%)	2,260 (26.8%)	-0.254	**<0.001**
Weight gain or loss of more than +/- 3 kg in the past year	2282 (17.7%)	1,845 (21.9%)	0.057	1.000
Do you want to improve your lifestyle habits such as exercise and diet?			0.037	1.000
I do not intend to improve (=1)	1,643 (12.7%)	957 (11.3%)		

	Non-DME<br>n = 2,368<br>12,927 tests	DME<br>n = 2,368<br>8,435 tests	Cox coefficient	Cox adjusted P value
I intend to improve (generally within 6 months) (=2)	3,220 (24.9%)	2,009 (23.8%)		
I intend to improve in the near future (generally within a month), and have begun to do so gradually (=3)	1,648 (12.7%)	1,006 (11.9%)		
Already working on improvement (less than 6 months) (=4)	1,288 (10.0%)	861 (10.2%)		
Already working on improvement (more than 6 months) (=5)	2,778 (21.5%)	1,707 (20.2%)		
If there is an opportunity to receive health guidance on lifestyle modification, would you use it?	3,244 (25.1%)	1,881 (22.3%)	-0.240	**<0.001**
