# Supplementary material for: Early Prediction of Diabetic Macular Edema via Machine Learning Survival Analysis on Checkup Data
Source: Ophthalmol Sci. 2026 Jun 1;6(8):101262. doi: 10.1016/j.xops.2026.101262 (PMC13355755; doi:10.1016/j.xops.2026.101262)
Supplement: Table S3 [file mmc3.pdf]

Table S3 Association of disease history variables with DME onset

Disease	chronic	ICD-10 block	ICD-10 code	Non-DME (incidence	Non-DME (%)	DME (incidence	DME (%)	coef	z_score	p_value	FDR
Gastritis and duodenitis	1	K00-K93	K29	2,943	22.77	1,659	19.67	0.99	17.44	4.03.E-68	1.22.E-65
Diabetic nephropathy	1	E00-E90	E14	1,071	8.28	1,298	15.39	1.28	17.18	3.80.E-66	1.15.E-63
Fatty liver	1	K00-K93	K76	2,131	16.48	1,121	13.29	1.03	13.32	1.70.E-40	5.10.E-38
Peripheral neuropathy	1	G00-G99	G62	1,409	10.90	685	8.12	1.02	12.10	1.00.E-33	2.98.E-31
Intraocular lens insertion	1	Z00-Z99	Z96	343	2.65	428	5.07	1.18	11.03	2.83.E-28	8.38.E-26
Chronic sinusitis	1	J00-J99	J32	1,125	8.70	476	5.64	0.99	10.24	1.38.E-24	4.07.E-22
Myopic astigmatism	0	H00-H59	H52	1,904	14.73	1,825	21.64	0.45	10.04	9.95.E-24	2.93.E-21
Hypercholesterolemia	0	E00-E90	E78	3,245	25.10	2,055	24.36	0.55	10.04	1.02.E-23	2.99.E-21
Hypertension	0	I00-I99	I10	5,418	41.91	3,519	41.72	0.48	9.53	1.53.E-21	4.47.E-19
Refractory gastroesophageal reflux disease requiring maintenance therap	1	K00-K93	K21	868	6.71	393	4.66	0.90	8.90	5.44.E-19	1.58.E-16
Vitreous hemorrhage	0	H00-H59	H43	40	0.31	144	1.71	1.29	8.78	1.70.E-18	4.92.E-16
Dyslipidemia	0	E00-E90	E78	1,584	12.25	913	10.82	0.53	8.27	1.33.E-16	3.82.E-14
Chronic heart failure	1	I00-I99	I50	576	4.46	312	3.70	1.01	8.10	5.71.E-16	1.63.E-13
Renal anemia	0	N00-N99	N19	71	0.55	149	1.77	1.32	7.36	1.85.E-13	5.26.E-11
Proliferative diabetic retinopathy	0	E00-E90	E14	30	0.23	173	2.05	1.04	7.31	2.73.E-13	7.72.E-11
Chronic renal failure	1	N00-N99	N18	268	2.07	222	2.63	1.27	7.17	7.46.E-13	2.10.E-10
Cataract	0	H00-H59	H26	469	3.63	622	7.37	0.50	7.06	1.69.E-12	4.76.E-10
Dry eye	0	H00-H59	H04	398	3.08	313	3.71	0.65	6.70	2.16.E-11	6.02.E-09
Macular edema	0	H00-H59	H35	73	0.56	218	2.58	0.82	6.47	9.84.E-11	2.74.E-08
Hyperphosphatemia	0	E00-E90	E83	42	0.32	87	1.03	1.48	6.41	1.47.E-10	4.08.E-08
Allergic rhinitis	0	J00-J99	J30	2,954	22.85	1,585	18.79	0.27	6.00	1.99.E-09	5.50.E-07
Hyperuricemia	0	E00-E90	E79	1,897	14.67	872	10.34	0.45	5.92	3.14.E-09	8.62.E-07
Hyperopia with astigmatism	0	H00-H59	H52	314	2.43	433	5.13	0.50	5.61	2.04.E-08	5.59.E-06
Chronic conjunctivitis	1	H00-H59	H10	143	1.11	116	1.38	0.98	5.58	2.39.E-08	6.52.E-06
Mixed astigmatism	0	H00-H59	H52	159	1.23	209	2.48	0.61	5.35	8.88.E-08	2.41.E-05
Acute upper respiratory tract inflammation	0	J00-J99	J06	1,133	8.76	800	9.48	0.24	4.90	9.82.E-07	2.63.E-04
Hyperkalemia	0	E00-E90	E87	41	0.32	86	1.02	1.02	4.82	1.43.E-06	3.83.E-04
Type 2 diabetic peripheral neuropathy	0	E00-E90	E11	19	0.15	26	0.31	1.34	4.78	1.77.E-06	4.72.E-04
Bronchial asthma	0	J00-J99	J45	1,007	7.79	544	6.45	0.37	4.69	2.72.E-06	7.22.E-04
Iron deficiency anemia	0	D50-D89	D50	450	3.48	361	4.28	0.54	4.65	3.35.E-06	8.85.E-04
Diabetic peripheral neuropathy	0	E00-E90	E14	223	1.73	423	5.01	0.51	4.61	4.10.E-06	1.08.E-03
Ischemic heart disease	0	I00-I99	I25	89	0.69	70	0.83	0.98	4.58	4.54.E-06	1.19.E-03
Essential hypertension	0	I00-I99	I10	279	2.16	177	2.10	0.71	4.52	6.30.E-06	1.64.E-03
Chronic kidney disease	1	N00-N99	N18	99	0.77	77	0.91	1.11	4.18	2.93.E-05	7.56.E-03
Cortical age-related cataract	0	H00-H59	H25	14	0.11	27	0.32	1.27	4.17	3.02.E-05	7.75.E-03
Allergic conjunctivitis	0	H00-H59	H10	1,210	9.36	724	8.58	0.26	4.15	3.38.E-05	8.66.E-03
Keratitis	0	H00-H59	H16	42	0.32	52	0.62	0.90	4.12	3.80.E-05	9.68.E-03
Hypoalbuminemia	0	E00-E90	E88	27	0.21	29	0.34	1.22	3.99	6.58.E-05	1.67.E-02
Hypertension	0	H00-H59	H40	127	0.98	149	1.77	0.56	3.96	7.43.E-05	1.88.E-02
Age-related cataract	0	H00-H59	H25	112	0.87	160	1.90	0.53	3.96	7.64.E-05	1.92.E-02
Secondary hyperparathyroidism	0	E00-E90	E21	55	0.43	76	0.90	0.95	3.95	7.81.E-05	1.96.E-02
Proliferative diabetic retinopathy, type 2 diabetes	0	E00-E90	E11	2	0.02	30	0.36	1.40	3.87	1.11.E-04	2.76.E-02
Retinal pre-membrane	0	H00-H59	H35	52	0.40	53	0.63	0.82	3.86	1.16.E-04	2.88.E-02
Xerosis	0	L00-L99	L85	315	2.44	213	2.53	0.43	3.72	2.02.E-04	4.98.E-02
Type 2 diabetic nephropathy, stage 3	1	E00-E90	E11	22	0.17	41	0.49	0.96	3.64	2.69.E-04	6.60.E-02
Glaucoma	0	H00-H59	H40	396	3.06	268	3.18	0.39	3.55	3.86.E-04	9.42.E-02
Refractory reflux esophagitis	0	K00-K93	K21	296	2.29	262	3.11	0.42	3.51	4.46.E-04	1.08.E-01
Type 2 diabetic nephropathy, stage 2	0	E00-E90	E11	182	1.41	119	1.41	0.59	3.51	4.53.E-04	1.09.E-01
Astigmatism	0	H00-H59	H52	59	0.46	112	1.33	0.71	3.49	4.82.E-04	1.16.E-01
Angiogenic glaucoma	0	H00-H59	H40	4	0.03	23	0.27	1.74	3.46	5.44.E-04	1.30.E-01
Influenza B	0	J00-J99	J10	87	0.67	40	0.47	0.64	3.42	6.33.E-04	1.50.E-01
Insomnia	0	G00-G99	G47	1,159	8.97	740	8.77	0.26	3.41	6.44.E-04	1.52.E-01
Metabolic acidosis	0	E00-E90	E87	17	0.13	20	0.24	1.10	3.37	7.38.E-04	1.74.E-01
Hemorrhage in the fundus of the eye	0	H00-H59	H35	12	0.09	56	0.66	0.88	3.37	7.64.E-04	1.79.E-01
Conjunctivitis	0	H00-H59	H10	352	2.72	360	4.27	0.27	3.32	8.98.E-04	2.09.E-01
Post-stroke sequelae	1	I00-I99	I69	101	0.78	122	1.45	0.85	3.32	9.00.E-04	2.09.E-01
Angina pectoris	0	I00-I99	I20	1,017	7.87	689	8.17	0.28	3.28	1.04.E-03	2.40.E-01
Cerebral infarction	0	I00-I99	I63	225	1.74	190	2.25	0.47	3.25	1.17.E-03	2.69.E-01
Diabetic retinopathy	0	E00-E90	E11	27	0.21	53	0.63	0.78	3.24	1.21.E-03	2.76.E-01
Age-related macular degeneration	0	H00-H59	H35	17	0.13	36	0.43	1.03	3.21	1.35.E-03	3.05.E-01
Postoperative iridocyclitis	0	H00-H59	H59	4	0.03	40	0.47	0.76	3.15	1.65.E-03	3.69.E-01
Reflux esophagitis	0	K00-K93	K21	1,169	9.04	627	7.43	0.20	3.13	1.76.E-03	3.92.E-01
Vertigo	0	R00-R99	R42	176	1.36	115	1.36	0.43	3.12	1.81.E-03	3.99.E-01
Schizophrenia	0	F00-F99	F20	140	1.08	70	0.83	0.74	3.10	1.96.E-03	4.31.E-01
Depression	0	F00-F99	F32	480	3.71	279	3.31	0.37	3.08	2.04.E-03	4.47.E-01
Sleep apnea syndrome	0	G00-G99	G47	573	4.43	254	3.01	0.41	3.08	2.05.E-03	4.48.E-01
Presbyopia	0	H00-H59	H52	377	2.92	354	4.20	0.27	3.04	2.37.E-03	5.10.E-01
Congestive heart failure	0	I00-I99	I50	208	1.61	124	1.47	0.50	2.95	3.22.E-03	6.84.E-01
Pain	0	R00-R99	R52	99	0.77	74	0.88	0.52	2.94	3.32.E-03	7.01.E-01
Seborrheic eczema	0	L00-L99	L85	295	2.28	189	2.24	0.32	2.92	3.50.E-03	7.36.E-01
Irritable bowel syndrome	0	K00-K93	K58	180	1.39	88	1.04	0.49	2.90	3.77.E-03	7.87.E-01
Post-cataract surgery	0	Z00-Z99	Z98	23	0.18	25	0.30	0.85	2.88	3.92.E-03	8.16.E-01
Arteriosclerosis obliterans	0	I00-I99	I70	255	1.97	226	2.68	0.41	2.82	4.81.E-03	9.95.E-01
Acute bronchitis	0	J00-J99	J20	1,761	13.62	1,050	12.45	0.11	2.53	1.13.E-02	1.00.E+00
Hyperlipidemia	0	E00-E90	E78	2,818	21.80	2,055	24.36	(0.03)	-0.61	5.40.E-01	1.00.E+00
Constipation	0	K00-K93	K59	881	6.82	722	8.56	0.22	2.67	7.66.E-03	1.00.E+00
Low back pain	0	M00-M99	M54	1,285	9.94	841	9.97	0.05	0.83	4.04.E-01	1.00.E+00
Gastric ulcer	0	K00-K93	K25	1,058	8.18	680	8.06	(0.10)	-1.36	1.74.E-01	1.00.E+00
Eczema	0	L00-L99	L30	642	4.97	415	4.92	0.05	0.74	4.61.E-01	1.00.E+00
Gastritis	0	K00-K93	K29	683	5.28	407	4.83	0.09	1.27	2.03.E-01	1.00.E+00
Acute pharyngitis	0	J00-J99	J02	676	5.23	359	4.26	0.15	2.10	3.54.E-02	1.00.E+00
Dehydration	0	E00-E90	E86	302	2.34	221	2.62	0.21	2.26	2.40.E-02	1.00.E+00
Pharyngitis	0	J00-J99	J02	478	3.70	288	3.41	0.14	1.77	7.59.E-02	1.00.E+00
Common cold	0	J00-J99	J00	445	3.44	261	3.09	0.16	2.00	4.53.E-02	1.00.E+00
Periarthritis scapulohumeralis	0	M00-M99	M75	587	4.54	377	4.47	0.05	0.68	4.95.E-01	1.00.E+00
Acute pharyngolaryngitis	0	J00-J99	J06	772	5.97	385	4.56	0.07	0.98	3.29.E-01	1.00.E+00
Headache	0	R00-R99	R51	406	3.14	256	3.03	0.25	2.64	8.18.E-03	1.00.E+00
Acute gastritis	0	K00-K93	K29	417	3.23	222	2.63	(0.02)	-0.23	8.14.E-01	1.00.E+00
Tinea pedis	0	A00-B99	B35	497	3.84	336	3.98	0.04	0.49	6.21.E-01	1.00.E+00
Bronchitis	0	J00-J99	J40	359	2.78	276	3.27	0.10	1.29	1.97.E-01	1.00.E+00
Diarrhea	0	A00-B99	A09	252	1.95	167	1.98	0.23	2.20	2.78.E-02	1.00.E+00
Osteoarthritis of the knee	0	M00-M99	M17	483	3.74	235	2.79	(0.06)	-0.54	5.90.E-01	1.00.E+00
Vomiting	0	R00-R99	R11	193	1.49	117	1.39	0.33	2.76	5.84.E-03	1.00.E+00
Acute sinusitis	0	J00-J99	J01	473	3.66	201	2.38	0.03	0.26	7.96.E-01	1.00.E+00
Liver dysfunction	0	K00-K93	K76	700	5.42	401	4.75	0.02	0.27	7.87.E-01	1.00.E+00
Influenza A	0	J00-J99	J10	254	1.96	121	1.43	0.26	2.46	1.40.E-02	1.00.E+00
Eyestrain	0	H00-H59	H53	212	1.64	234	2.77	0.10	0.86	3.92.E-01	1.00.E+00
Osteoporosis	0	M00-M99	M81	241	1.86	170	2.02	0.20	1.24	2.14.E-01	1.00.E+00
Myopia	0	H00-H59	H52	384	2.97	271	3.21	0.20	2.14	3.21.E-02	1.00.E+00
Skin infection	0	L00-L99	L08	146	1.13	99	1.17	0.29	2.07	3.88.E-02	1.00.E+00
Posterior cataract	0	H00-H59	H26	32	0.25	57	0.68	0.45	1.90	5.69.E-02	1.00.E+00
Acute gastroenteritis	0	A00-B99	A09	210	1.62	151	1.79	0.08	0.83	4.08.E-01	1.00.E+00
Corneal erosion	0	H00-H59	H16	126	0.97	122	1.45	0.30	2.13	3.30.E-02	1.00.E+00
Blepharitis	0	H00-H59	H01	107	0.83	77	0.91	0.36	2.10	3.57.E-02	1.00.E+00
Heart failure	0	I00-I99	I50	315	2.44	136	1.61	(0.13)	-0.85	3.93.E-01	1.00.E+00
Colon polyp	0	K00-K93	K63	302	2.34	125	1.48	0.07	0.47	6.35.E-01	1.00.E+00
Dry keratoconjunctivitis	0	H00-H59	H16	208	1.61	132	1.56	0.37	2.43	1.50.E-02	1.00.E+00
Peripheral neuropathic pain	0	G00-G99	G64	169	1.31	138	1.64	0.38	2.58	9.79.E-03	1.00.E+00
Cervico-omo-brachial syndrome	0	M00-M99	M53	399	3.09	227	2.69	(0.00)	-0.03	9.72.E-01	1.00.E+00
Contact dermatitis	0	L00-L99	L25	166	1.28	124	1.47	(0.14)	-1.11	2.65.E-01	1.00.E+00
Lumbar spinal canal stenosis	0	M00-M99	M48	243	1.88	135	1.60	0.29	2.22	2.62.E-02	1.00.E+00
Pruritus cutaneus	0	L00-L99	L29	131	1.01	89	1.06	0.15	0.85	3.95.E-01	1.00.E+00
Influenza	0	J00-J99	J11	166	1.28	98	1.16	0.13	1.07	2.86.E-01	1.00.E+00
Degenerative lumbar disease	0	M00-M99	M47	189	1.46	123	1.46	0.25	1.81	7.05.E-02	1.00.E+00
Carotid artery stenosis	0	I00-I99	I65	170	1.32	110	1.30	0.34	1.99	4.68.E-02	1.00.E+00
Cervical spondylosis	0	M00-M99	M47	273	2.11	143	1.70	(0.03)	-0.24	8.12.E-01	1.00.E+00
Helicobacter pylori infection	0	A00-B99	A49	212	1.64	125	1.48	0.03	0.26	7.94.E-01	1.00.E+00
Urticaria	0	L00-L99	L50	271	2.10	143	1.70	0.23	1.86	6.33.E-02	1.00.E+00
Acute enteritis	0	A00-B99	A09	130	1.01	82	0.97	(0.04)	-0.28	7.76.E-01	1.00.E+00
Cystitis	0	N00-N99	N30	112	0.87	60	0.71	(0.11)	-0.58	5.62.E-01	1.00.E+00
Chronic bronchitis	0	J00-J99	J42	222	1.72	139	1.65	0.12	0.80	4.24.E-01	1.00.E+00

Cellulitis	0	L00-L99	L03	73	0.56	106	1.26	0.08	0.58	5.61.E-01	1.00.E+00
Neuropathic pain	0	G00-G99	G98	146	1.13	90	1.07	0.39	2.48	1.30.E-02	1.00.E+00
Enlarged prostate	0	N00-N99	N40	384	2.97	164	1.94	0.10	0.77	4.40.E-01	1.00.E+00
Atrophic gastritis	0	K00-K93	K29	196	1.52	114	1.35	0.23	1.46	1.43.E-01	1.00.E+00
Fever	0	R00-R99	R50	124	0.96	50	0.59	0.36	1.97	4.89.E-02	1.00.E+00
Arrhythmia	0	I00-I99	I49	289	2.24	120	1.42	(0.23)	-1.32	1.85.E-01	1.00.E+00
End-stage renal failure	0	N00-N99	N18	22	0.17	30	0.36	0.31	0.96	3.37.E-01	1.00.E+00
Earwax plug	0	H60-H95	H61	142	1.10	75	0.89	0.01	0.08	9.35.E-01	1.00.E+00
Anxiety neurosis	0	F00-F99	F41	234	1.81	140	1.66	0.32	2.09	3.64.E-02	1.00.E+00
Senile cataract	0	H00-H59	H25	14	0.11	36	0.43	0.86	2.70	7.01.E-03	1.00.E+00
Punctate superficial keratitis	0	H00-H59	H16	67	0.52	59	0.70	0.20	0.92	3.57.E-01	1.00.E+00
Angina pectoris	0	I00-I99	I20	110	0.85	80	0.95	0.47	2.35	1.87.E-02	1.00.E+00
Athlete's foot	0	A00-B99	B35	136	1.05	87	1.03	0.31	1.89	5.89.E-02	1.00.E+00
Arteriosclerosis obliterans of the lower extremities	0	I00-I99	I70	91	0.70	81	0.96	0.51	2.44	1.46.E-02	1.00.E+00
Acute tonsillitis	0	J00-J99	J03	190	1.47	94	1.11	0.30	2.24	2.48.E-02	1.00.E+00
Decreased kidney function	0	N00-N99	N28	196	1.52	106	1.26	0.42	2.67	7.51.E-03	1.00.E+00
Muscle pain	0	M00-M99	M79	205	1.59	110	1.30	(0.03)	-0.21	8.35.E-01	1.00.E+00
Dermatitis	0	L00-L99	L30	103	0.80	99	1.17	0.12	0.77	4.44.E-01	1.00.E+00
Acute conjunctivitis	0	H00-H59	H10	70	0.54	40	0.47	0.20	0.93	3.54.E-01	1.00.E+00
Tonsillitis	0	J00-J99	J03	156	1.21	116	1.38	0.25	2.00	4.55.E-02	1.00.E+00
Lumbar disc hernia	0	M00-M99	M51	194	1.50	109	1.29	0.11	0.74	4.59.E-01	1.00.E+00
Lumbar disc disease	0	M00-M99	M51	219	1.69	137	1.62	0.14	1.16	2.46.E-01	1.00.E+00
Urinary tract infection	0	N00-N99	N39	73	0.56	47	0.56	0.17	0.89	3.73.E-01	1.00.E+00
Infectious gastroenteritis	0	A00-B99	A09	123	0.95	78	0.92	0.20	1.44	1.49.E-01	1.00.E+00
Pneumonia	0	J00-J99	J18	67	0.52	42	0.50	0.17	0.82	4.11.E-01	1.00.E+00
Chronic hepatitis	0	K00-K93	K73	368	2.85	249	2.95	(0.02)	-0.14	8.91.E-01	1.00.E+00
Hypothyroidism	0	E00-E90	E03	185	1.43	103	1.22	0.43	1.74	8.16.E-02	1.00.E+00
Refractive error	0	H00-H59	H52	73	0.56	90	1.07	0.46	2.19	2.88.E-02	1.00.E+00
Type 2 diabetic nephropathy	0	E00-E90	E11	53	0.41	67	0.79	0.64	2.75	5.89.E-03	1.00.E+00
Pharyngolaryngitis	0	J00-J99	J06	206	1.59	91	1.08	0.11	0.69	4.91.E-01	1.00.E+00
Seborrheic dermatitis	0	L00-L99	L21	158	1.22	109	1.29	(0.13)	-0.82	4.12.E-01	1.00.E+00
Dizziness	0	R00-R99	R42	112	0.87	78	0.92	0.31	1.76	7.85.E-02	1.00.E+00
Cholelithiasis	0	K00-K93	K80	259	2.00	152	1.80	0.04	0.26	7.98.E-01	1.00.E+00
Carotid artery sclerosis	0	I00-I99	I65	162	1.25	117	1.39	0.33	1.78	7.49.E-02	1.00.E+00
Atopic dermatitis	0	L00-L99	L20	261	2.02	150	1.78	0.34	2.31	2.08.E-02	1.00.E+00
Respiratory failure	0	J00-J99	J96	40	0.31	29	0.34	0.70	2.75	5.97.E-03	1.00.E+00
Chronic myocardial infarction	0	I00-I99	I25	235	1.82	165	1.96	0.23	1.15	2.51.E-01	1.00.E+00
Stomatitis	0	K00-K93	K12	127	0.98	88	1.04	(0.26)	-1.54	1.24.E-01	1.00.E+00
Sensorineural hearing loss	0	H60-H95	H90	72	0.56	58	0.69	0.23	1.22	2.21.E-01	1.00.E+00
Enlargement of optic disc cup	0	H00-H59	H40	128	0.99	87	1.03	0.34	2.07	3.86.E-02	1.00.E+00
Callus	0	L00-L99	L84	53	0.41	77	0.91	0.43	2.45	1.43.E-02	1.00.E+00
Otitis externa	0	H60-H95	H60	96	0.74	79	0.94	0.03	0.15	8.79.E-01	1.00.E+00
Uveitis	0	H00-H59	H20	40	0.31	60	0.71	0.55	2.63	8.61.E-03	1.00.E+00
Diabetic neuropathy	0	E00-E90	E14	55	0.43	109	1.29	0.32	1.48	1.39.E-01	1.00.E+00
Macular degeneration	0	H00-H59	H35	64	0.50	80	0.95	0.48	2.47	1.35.E-02	1.00.E+00
Intraoperative hypertension	0	S00-T98	T81	17	0.13	15	0.18	0.34	0.88	3.80.E-01	1.00.E+00
Infectious enteritis	0	A00-B99	A09	114	0.88	48	0.57	0.36	2.04	4.09.E-02	1.00.E+00
Chronic eczema	0	L00-L99	L30	139	1.08	65	0.77	0.28	1.58	1.13.E-01	1.00.E+00
Verruca Vulgaris	0	A00-B99	B07	145	1.12	57	0.68	0.12	0.67	5.03.E-01	1.00.E+00
Cramp in the Leg	0	R00-R99	R25	84	0.65	63	0.75	0.34	1.85	6.43.E-02	1.00.E+00
Gout	0	M00-M99	M10	393	3.04	173	2.05	0.03	0.23	8.15.E-01	1.00.E+00
Proteinuria	0	R00-R99	R80	140	1.08	126	1.49	0.48	2.72	6.47.E-03	1.00.E+00
Eczema of the Outer Ear	0	H60-H95	H60	144	1.11	51	0.60	0.02	0.11	9.10.E-01	1.00.E+00
Edema	0	R00-R99	R60	77	0.60	52	0.62	0.50	2.30	2.12.E-02	1.00.E+00
Skin ulcer	0	L00-L99	L98	35	0.27	32	0.38	(0.24)	-0.80	4.24.E-01	1.00.E+00
Shingles	0	A00-B99	B02	92	0.71	48	0.57	(0.06)	-0.29	7.72.E-01	1.00.E+00
Arteriosclerosis	0	I00-I99	I70	157	1.21	108	1.28	0.28	1.63	1.03.E-01	1.00.E+00
Abdominal pain	0	R00-R99	R10	95	0.73	52	0.62	0.21	1.14	2.54.E-01	1.00.E+00
Chest pain	0	R00-R99	R07	62	0.48	61	0.72	0.05	0.33	7.39.E-01	1.00.E+00
Degenerative cervical spondylosis	0	M00-M99	M47	115	0.89	74	0.88	0.13	0.72	4.70.E-01	1.00.E+00
Neurotic disorder	0	F00-F99	F48	222	1.72	104	1.23	0.48	2.69	7.04.E-03	1.00.E+00
Tinea unguium	0	A00-B99	B35	71	0.55	63	0.75	0.17	0.83	4.06.E-01	1.00.E+00
Habitual constipation	0	K00-K93	K59	36	0.28	38	0.45	0.63	2.24	2.51.E-02	1.00.E+00
Cervical erosion	0	N00-N99	N86	61	0.47	51	0.60	0.11	0.49	6.21.E-01	1.00.E+00
Nephrotic syndrome	0	N00-N99	N04	34	0.26	64	0.76	0.63	2.51	1.21.E-02	1.00.E+00
Overactive bladder	0	N00-N99	N32	167	1.29	79	0.94	0.44	2.11	3.46.E-02	1.00.E+00
Chronic pain	0	R00-R99	R52	106	0.82	35	0.41	0.41	1.60	1.09.E-01	1.00.E+00
Obesity	0	E00-E90	E66	217	1.68	135	1.60	0.23	1.36	1.73.E-01	1.00.E+00
Hordeolum	0	H00-H59	H00	76	0.59	58	0.69	0.09	0.53	5.94.E-01	1.00.E+00
Myopia	0	H00-H59	H52	106	0.82	73	0.87	0.35	1.70	8.88.E-02	1.00.E+00
Acute respiratory failure	0	J00-J99	J96	14	0.11	15	0.18	0.57	1.52	1.29.E-01	1.00.E+00
Unstable angina pectoris	0	I00-I99	I20	110	0.85	54	0.64	0.29	1.24	2.16.E-01	1.00.E+00
Retinal vein occlusion	0	H00-H59	H34	30	0.23	43	0.51	0.58	1.90	5.73.E-02	1.00.E+00
Liver damage	0	K00-K93	K76	235	1.82	99	1.17	0.16	0.91	3.64.E-01	1.00.E+00
Gastroenteritis	0	A00-B99	A09	97	0.75	45	0.53	0.17	0.94	3.49.E-01	1.00.E+00
Acute cystitis	0	N00-N99	N30	43	0.33	37	0.44	0.29	1.31	1.90.E-01	1.00.E+00
Mitral valve insufficiency	0	I00-I99	I34	132	1.02	58	0.69	0.37	1.60	1.09.E-01	1.00.E+00
Ureteral calculus	0	N00-N99	N20	202	1.56	121	1.43	0.07	0.47	6.38.E-01	1.00.E+00
Type 2 diabetic nephropathy, stage 1	0	E00-E90	E11	145	1.12	79	0.94	0.40	1.97	4.88.E-02	1.00.E+00
Hypokalemia	0	E00-E90	E87	47	0.36	35	0.41	0.46	1.55	1.20.E-01	1.00.E+00
Gastric polyp	0	K00-K93	K31	129	1.00	50	0.59	0.09	0.43	6.70.E-01	1.00.E+00
Disuse syndrome	0	M00-M99	M62	12	0.09	10	0.12	0.47	0.96	3.36.E-01	1.00.E+00
Postoperative pain	0	S00-T98	T88	28	0.22	22	0.26	0.35	1.06	2.89.E-01	1.00.E+00
Iritis	0	H00-H59	H20	16	0.12	28	0.33	0.93	2.09	3.68.E-02	1.00.E+00
Uterine myoma	0	C00-D48	D25	100	0.77	48	0.57	0.12	0.55	5.80.E-01	1.00.E+00
Renal calculus	0	N00-N99	N20	237	1.83	126	1.49	0.25	1.50	1.33.E-01	1.00.E+00
Anorexia	0	R00-R99	R63	67	0.52	46	0.55	0.19	1.00	3.18.E-01	1.00.E+00
Coronary artery stent implantation	0	Z00-Z99	Z95	181	1.40	88	1.04	0.23	1.21	2.25.E-01	1.00.E+00
Exudative otitis media	0	H60-H95	H65	99	0.77	47	0.56	(0.20)	-0.98	3.25.E-01	1.00.E+00
Dialysis shunt stenosis	0	S00-T98	T82	7	0.05	17	0.20	0.90	2.30	2.17.E-02	1.00.E+00
Hematuria	0	R00-R99	R31	117	0.91	43	0.51	(0.11)	-0.57	5.71.E-01	1.00.E+00
Osteoarthritis	0	M00-M99	M47	122	0.94	81	0.96	(0.12)	-0.63	5.32.E-01	1.00.E+00
Hyperopia	0	H00-H59	H52	75	0.58	68	0.81	0.25	1.18	2.39.E-01	1.00.E+00
Subconjunctival hemorrhage	0	H00-H59	H11	52	0.40	34	0.40	(0.12)	-0.58	5.63.E-01	1.00.E+00
Acute laryngitis	0	J00-J99	J04	114	0.88	47	0.56	(0.30)	-1.57	1.17.E-01	1.00.E+00
Neurogenic bladder	0	N00-N99	N31	65	0.50	49	0.58	0.70	2.73	6.41.E-03	1.00.E+00
Foreign body in the conjunctiva	0	S00-T98	T15	45	0.35	38	0.45	0.31	1.43	1.54.E-01	1.00.E+00
Ingrown toenail	0	L00-L99	L60	39	0.30	41	0.49	0.33	1.50	1.33.E-01	1.00.E+00
Atrial fibrillation	0	I00-I99	I48	164	1.27	90	1.07	0.10	0.44	6.62.E-01	1.00.E+00
Pleural effusion	0	J00-J99	J90	16	0.12	10	0.12	0.74	1.25	2.12.E-01	1.00.E+00
Folliculitis	0	L00-L99	L73	61	0.47	46	0.55	(0.24)	-1.01	3.12.E-01	1.00.E+00
Wound infection	0	S00-T98	T79	20	0.15	23	0.27	0.39	1.28	2.02.E-01	1.00.E+00
Sprained ankle	0	S00-T98	S93	51	0.39	35	0.41	0.02	0.08	9.38.E-01	1.00.E+00
Trichiasis	0	H00-H59	H02	66	0.51	41	0.49	0.10	0.41	6.84.E-01	1.00.E+00
Nausea	0	R00-R99	R11	38	0.29	23	0.27	0.35	1.34	1.81.E-01	1.00.E+00
Internal hemorrhoids	0	K00-K93	K64	163	1.26	45	0.53	(0.19)	-0.79	4.32.E-01	1.00.E+00
Cervical radiculopathy	0	M00-M99	M47	83	0.64	67	0.79	0.17	0.89	3.74.E-01	1.00.E+00
Ménière's disease	0	H60-H95	H81	103	0.80	49	0.58	0.23	1.09	2.74.E-01	1.00.E+00
Diffuse superficial keratitis	0	H00-H59	H16	56	0.43	61	0.72	0.48	2.37	1.76.E-02	1.00.E+00
Rheumatoid arthritis	0	M00-M99	M06	190	1.47	73	0.87	0.38	1.52	1.29.E-01	1.00.E+00
Secondary glaucoma	0	H00-H59	H40	5	0.04	35	0.41	0.81	2.67	7.64.E-03	1.00.E+00
Depression	0	F00-F99	F32	124	0.96	62	0.74	0.22	0.95	3.44.E-01	1.00.E+00
Renal edema	0	R00-R99	R60	34	0.26	14	0.17	1.04	2.79	5.28.E-03	1.00.E+00
Hay fever	0	J00-J99	J30	163	1.26	89	1.06	0.06	0.33	7.38.E-01	1.00.E+00
Allergic dermatitis	0	L00-L99	L27	62	0.48	37	0.44	(0.04)	-0.18	8.56.E-01	1.00.E+00
Eczema of the hands	0	L00-L99	L30	94	0.73	49	0.58	0.26	1.27	2.05.E-01	1.00.E+00
Corn	0	L00-L99	L84	53	0.41	41	0.49	0.44	1.93	5.38.E-02	1.00.E+00
Chronic arterial obstruction	0	I00-I99	I74	21	0.16	59	0.70	0.16	0.68	4.95.E-01	1.00.E+00
Tinea	0	A00-B99	B35	69	0.53	65	0.77	0.07	0.39	6.99.E-01	1.00.E+00
Asthmatic bronchitis	0	J00-J99	J45	89	0.69	55	0.65	(0.18)	-1.01	3.14.E-01	1.00.E+00
Gallbladder polyp	0	K00-K93	K82	179	1.38	52	0.62	0.08	0.35	7.26.E-01	1.00.E+00
Sciatica	0	M00-M99	M54	64	0.50	49	0.58	(0.09)	-0.39	6.98.E-01	1.00.E+00
Vitreous opacity	0	H00-H59	H43	49	0.38	37	0.44	(0.17)	-0.54	5.87.E-01	1.00.E+00

Rib fracture	0	S00-T98	S22	39	0.30	23	0.27	0.44	1.69	9.18.E-02	1.00.E+00
Skin erosion	0	L00-L99	L98	44	0.34	24	0.28	0.51	1.82	6.86.E-02	1.00.E+00
Acute rhinitis	0	J00-J99	J00	112	0.87	32	0.38	0.16	0.73	4.63.E-01	1.00.E+00
Abnormal shadow in the chest	0	R00-R99	R91	105	0.81	36	0.43	0.30	1.41	1.58.E-01	1.00.E+00
Acute heart failure	0	I00-I99	I50	10	0.08	19	0.23	(0.34)	-1.08	2.82.E-01	1.00.E+00
open-angle glaucoma	0	H00-H59	H40	55	0.43	27	0.32	0.50	1.20	2.29.E-01	1.00.E+00
renal cyst	0	N00-N99	N28	109	0.84	42	0.50	0.14	0.54	5.86.E-01	1.00.E+00
Helicobacter pylori gastritis	0	K00-K93	K29	92	0.71	55	0.65	0.28	1.47	1.41.E-01	1.00.E+00
Peripheral neuritis	0	G00-G99	G62	92	0.71	41	0.49	(0.37)	-1.51	1.31.E-01	1.00.E+00
Hyperthyroidism	0	E00-E90	E05	160	1.24	62	0.74	(0.44)	-1.36	1.75.E-01	1.00.E+00
Normal tension glaucoma	0	H00-H59	H40	114	0.88	56	0.66	0.27	1.06	2.91.E-01	1.00.E+00
Peripheral circulatory disturbance	0	I00-I99	I73	63	0.49	32	0.38	0.09	0.29	7.75.E-01	1.00.E+00
Hypoxemia	0	R00-R99	R09	19	0.15	24	0.28	(0.01)	-0.04	9.66.E-01	1.00.E+00
Acute otitis externa	0	H60-H95	H60	38	0.29	31	0.37	0.14	0.62	5.36.E-01	1.00.E+00
Tinnitus	0	H60-H95	H93	62	0.48	29	0.34	0.10	0.42	6.74.E-01	1.00.E+00
Acute circulatory failure	0	R00-R99	R57	16	0.12	9	0.11				
Keratosis palmaris et plantaris	0	L00-L99	L85	59	0.46	30	0.36				
Lateral epicondylitis	0	M00-M99	M77	85	0.66	57	0.68				
Atheromatous pyorrhea	0	L00-L99	L72	27	0.21	24	0.28				
Acute lumbago	0	M00-M99	M54	35	0.27	27	0.32				
Bilateral osteoarthritis of the knee	0	M00-M99	M17	93	0.72	48	0.57				
Ventricular extrasystole	0	I00-I99	I49	177	1.37	73	0.87				
Eustachian tube stenosis	0	H60-H95	H68	39	0.30	27	0.32				
Tension-type headache	0	G00-G99	G44	86	0.67	67	0.79				
Migraine	0	G00-G99	G43	136	1.05	57	0.68				
Bleeding tendency	0	D50-D89	D69	26	0.20	21	0.25				
Goiter	0	E00-E90	E04	112	0.87	39	0.46				
Chronic cerebral infarction	0	I00-I99	I69	20	0.15	39	0.46				
Acute abdomen	0	R00-R99	R10	32	0.25	18	0.21				
Renal failure	0	N00-N99	N19	33	0.26	16	0.19				
Vitamin deficiency	0	E00-E90	E56	38	0.29	28	0.33				
Seborrheic keratosis	0	L00-L99	L82	101	0.78	28	0.33				
Lumbar spondylosis	0	M00-M99	M47	66	0.51	41	0.49				
Cough	0	R00-R99	R05	44	0.34	26	0.31				
Hypocalcemia	0	E00-E90	E83	22	0.17	32	0.38				
Knee joint pain	0	M00-M99	M25	53	0.41	34	0.40				
Superficial keratitis	0	H00-H59	H16	51	0.39	30	0.36				
Chronic laryngitis	0	J00-J99	J37	132	1.02	40	0.47				
Plantar keratosis	0	Q00-Q99	Q82	36	0.28	37	0.44				
Osteoarthritis of the hip	0	M00-M99	M16	64	0.50	33	0.39				
Osteoarthritis	0	M00-M99	M19	75	0.58	32	0.38				
Thyroid tumor	0	C00-D48	D44	91	0.70	39	0.46				
Hypertensive emergency	0	I00-I99	I10	16	0.12	6	0.07				
Myofascial low back pain	0	M00-M99	M54	64	0.50	37	0.44				
Retinal vein occlusion	0	H00-H59	H34	5	0.04	20	0.24				
Abdominal distension	0	R00-R99	R14	39	0.30	31	0.37				
Bacterial infection	0	A00-B99	A49	41	0.32	21	0.25				
Postoperative ocular hypertension	0	H00-H59	H59	3	0.02	11	0.13				
Proliferative vitreoretinopathy	0	H00-H59	H35	7	0.05	10	0.12				
Chronic glomerulonephritis	0	N00-N99	N03	97	0.75	74	0.88				
Tear secretion deficiency	0	H00-H59	H04	52	0.40	73	0.87				
Painful muscle spasm	0	R00-R99	R25	35	0.27	41	0.49				
Psychosomatic illness	0	F00-F99	F45	102	0.79	49	0.58				
Hemorrhoids	0	K00-K93	K64	79	0.61	31	0.37				
Stiff shoulders	0	M00-M99	M62	75	0.58	47	0.56				
Foot skin ulcer	0	L00-L99	L97	6	0.05	16	0.19				
Blepharitis	0	H00-H59	H01	38	0.29	24	0.28				
Posterior vitreous detachment	0	H00-H59	H43	69	0.53	40	0.47				
Sinusitis	0	J00-J99	J32	53	0.41	33	0.39				
Paronychia	0	L00-L99	L03	18	0.14	18	0.21				
Post-vitrectomy	0	Z00-Z99	Z98	6	0.05	8	0.09				
Acute pneumonia	0	J00-J99	J18	24	0.19	13	0.15				
Hip joint pain	0	M00-M99	M25	26	0.20	31	0.37				
Epilepsy	0	G00-G99	G40	79	0.61	33	0.39				
Acute pyelonephritis	0	N00-N99	N10	12	0.09	18	0.21				
Cervical disc herniation	0	M00-M99	M50	93	0.72	27	0.32				
Foot eczema	0	L00-L99	L30	44	0.34	28	0.33				
Foot arthritis	0	M00-M99	M13	49	0.38	38	0.45				
Back pain	0	M00-M99	M54	52	0.40	30	0.36				
Dialysis hypotension	0	S00-T98	T80	10	0.08	7	0.08				
Bilateral sensorineural hearing loss	0	H60-H95	H90	29	0.22	21	0.25				
Arteriosclerotic retinopathy	0	I00-I99	I70	42	0.32	53	0.63				
Lower limb edema	0	R00-R99	R60	28	0.22	27	0.32				
Chronic thyroiditis	0	E00-E90	E06	80	0.62	52	0.62				
Diabetic cataract	0	E00-E90	E14	7	0.05	43	0.51				
Hypertensive retinopathy	0	H00-H59	H35	47	0.36	22	0.26				
Sudden deafness	0	H60-H95	H91	32	0.25	17	0.20				
Acne vulgaris	0	L00-L99	L70	45	0.35	26	0.31				
Knee arthritis	0	M00-M99	M13	62	0.48	22	0.26				
Dialysis shunt occlusion	0	S00-T98	T82	4	0.03	11	0.13				
Edema of the lower leg	0	R00-R99	R60	6	0.05	18	0.21				
Bacterial conjunctivitis	0	A00-B99	B99	13	0.10	14	0.17				
Erosive gastritis	0	K00-K93	K29	59	0.46	44	0.52				
Arthritis of the wrist	0	M00-M99	M13	30	0.23	28	0.33				
Septicemia	0	A00-B99	A41	6	0.05	6	0.07				
Chalazion	0	H00-H59	H00	26	0.20	25	0.30				
Fecal occult blood	0	R00-R99	R19	29	0.22	19	0.23				
Myocardial infarction	0	I00-I99	I21	87	0.67	49	0.58				
Neuralgia	0	M00-M99	M79	52	0.40	25	0.30				
Carnitine deficiency	0	E00-E90	E71	5	0.04	22	0.26				
Duodenal ulcer	0	K00-K93	K26	119	0.92	62	0.74				
Pyelonephritis	0	N00-N99	N12	19	0.15	15	0.18				
Neck pain	0	M00-M99	M54	49	0.38	20	0.24				
Orthostatic hypotension	0	I00-I99	I95	8	0.06	15	0.18				
Eczema of the head	0	L00-L99	L30	52	0.40	29	0.34				
Atherosclerosis	0	L00-L99	L72	35	0.27	24	0.28				
Middle finger flexor tendinitis	0	M00-M99	M65	24	0.19	13	0.15				
Skin abscess	0	L00-L99	L02	21	0.16	15	0.18				
Gastric ulcer scar	0	K00-K93	K25	65	0.50	43	0.51				
Acute otitis media	0	H60-H95	H66	34	0.26	21	0.25				
Convulsions	0	R00-R99	R25	27	0.21	37	0.44				
Skin tumor	0	C00-D48	D48	32	0.25	24	0.28				
Hypertriglyceridemia	0	E00-E90	E78	151	1.17	91	1.08				
Abrasion	0	S00-T98	T14	17	0.13	14	0.17				
Eye pain	0	H00-H59	H57	8	0.06	17	0.20				
Chronic pancreatitis	0	K00-K93	K86	106	0.82	79	0.94				
Chronic kidney disease stage G5	0	N00-N99	N18	1	0.01	5	0.06				
Retinal detachment due to hiatal hernia	0	H00-H59	H33	23	0.18	31	0.37				
Head injury	0	S00-T98	S00	16	0.12	9	0.11				
Nasal hemorrhage	0	R00-R99	R04	48	0.37	17	0.20				
Generalized eczema	0	L00-L99	L30	31	0.24	22	0.26				
Shoulder joint pain syndrome	0	M00-M99	M25	36	0.28	32	0.38				
Deep vein thrombosis	0	I00-I99	I80	46	0.36	17	0.20				
Retinal hemorrhage	0	H00-H59	H35	22	0.17	20	0.24				
Lower limb pain	0	M00-M99	M79	18	0.14	11	0.13				
Autonomic neuropathy	0	G00-G99	G90	75	0.58	59	0.70				
Joint pain	0	M00-M99	M25	45	0.35	21	0.25				
Cervical sprain	0	S00-T98	S13	34	0.26	15	0.18				
Keratoconjunctivitis	0	H00-H59	H16	14	0.11	10	0.12				
Cardiac hypertrophy	0	I00-I99	I51	41	0.32	47	0.56				
Blood clotting disorder	0	D50-D89	D68	33	0.26	18	0.21				
Impetigo	0	L00-L99	L01	23	0.18	27	0.32				
Hypoglycemia	0	E00-E90	E16	11	0.09	26	0.31				

Leg skin ulcer	0	L00-L99	L97	9	0.07	17	0.20
Chronic cystitis	0	N00-N99	N30	49	0.38	33	0.39
Cardiac valvular disease	0	I00-I99	I38	43	0.33	39	0.46
Knee contusion	0	S00-T98	S80	21	0.16	13	0.15
Adjustive asthenopia	0	H00-H59	H52	42	0.32	18	0.21
Herpes simplex	0	A00-B99	B00	43	0.33	24	0.28
Chronic enteritis	0	K00-K93	K52	57	0.44	19	0.23
Menopausal syndrome	0	N00-N99	N95	52	0.40	47	0.56
Peripheral vertigo	0	H60-H95	H81	18	0.14	16	0.19
Chest contusion	0	S00-T98	S20	11	0.09	13	0.15
Lacunar infarction	0	I00-I99	I63	14	0.11	36	0.43
Ischemic cerebrovascular disease	0	I00-I99	I67	60	0.46	31	0.37
Vulvo-vaginal candidiasis	0	A00-B99	B37	20	0.15	22	0.26
Dyspnea	0	R00-R99	R06	13	0.10	9	0.11
Bacterial vaginosis	0	N00-N99	N76	19	0.15	13	0.15
Pollakisuria	0	R00-R99	R35	40	0.31	24	0.28
Hypoglycemic attack	0	E00-E90	E16	8	0.06	18	0.21
Iritis-iridocyclitis	0	H00-H59	H20	4	0.03	4	0.05
Posterior subcapsular cataract	0	H00-H59	H25	5	0.04	13	0.15
Diabetic gangrene	0	E00-E90	E14	-	-	10	0.12
Carpal tunnel syndrome	0	G00-G99	G56	27	0.21	18	0.21
Chemotherapy-induced nausea and vomiting	0	R00-R99	R11	16	0.12	11	0.13
Progressive palmar keratosis	0	L00-L99	L25	26	0.20	13	0.15
Abnormal lipid metabolism	0	E00-E90	E78	96	0.74	64	0.76
Cutaneous abscess, furuncle and carbuncle	0	L00-L99	L02	25	0.19	13	0.15
Consciousness disorder	0	R00-R99	R40	6	0.05	5	0.06
Type 2 diabetes mellitus with neurological complications	0	E00-E90	E11	4	0.03	31	0.37
Retinal hole	0	H00-H59	H33	41	0.32	18	0.21
Presbyopia cataract	0	H00-H59	H26	10	0.08	16	0.19
Toe skin ulcer	0	L00-L99	L97	6	0.05	7	0.08
Acanthosis nigricans	0	S00-T98	T63	22	0.17	17	0.20
MRSA infection	0	A00-B99	A49	4	0.03	5	0.06
Hyperkeratosis	0	L00-L99	L85	25	0.19	20	0.24
Acute nasopharyngitis	0	J00-J99	J00	32	0.25	23	0.27
Purulent skin disease	0	L00-L99	L08	28	0.22	8	0.09
Type 2 diabetic nephropathy, stage 4	0	E00-E90	E11	11	0.09	15	0.18
Tachyarrhythmia	0	R00-R99	R00	45	0.35	31	0.37
