# Supplementary material for: Early Prediction of Diabetic Macular Edema via Machine Learning Survival Analysis on Checkup Data
Source: Ophthalmol Sci. 2026 Jun 1;6(8):101262. doi: 10.1016/j.xops.2026.101262 (PMC13355755; doi:10.1016/j.xops.2026.101262)
Supplement: Table S4 [file mmc4.pdf]

Table S4 Performance metrics of the survival models predicting DME onset

Method	C-index	C-index difference	IBS	mean AUC
Chronic gastritis[a]	.498 (.498–.502)	.195 (.190–.199)	.219 (.219–.220)	.521 (.479–.521)
Diabetic nephropathy[a]	.529 (.529–.529)	.165 (.160–.169)	.217 (.216–.218)	.537 (.537–.537)
Diabetes medication[a]	.601 (.601–.601)	.093 (.088–.097)	.208 (.208–.209)	.618 (.618–.618)
Urinary sugar[a]	.578 (.578–.578)	.116 (.110–.119)	.210 (.209–.211)	.605 (.605–.605)
Fatty liver[a]	.491 (.491–.509)	.203 (.182–.207)	.220 (.219–.221)	.497 (.497–.503)
HbA1c[a]	.638 (.638–.638)	.056 (.050–.059)	.207 (.206–.207)	.668 (.668–.668)
Multivariate Cox	.665 (.655–.674)	.029 (.019–.038)	.191 (.186–.197)	.727 (.713–.740)
Multivariate Cox LASSO	.679 (.671–.686)	.015 (.005–.022)	.183 (.179–.187)	.745 (.732–.755)
Multivariate Cox RIDGE	.675 (.667–.686)	.019 (.007–.026)	.185 (.181–.188)	.739 (.728–.751)
Multivariate CoxNet	.679 (.670–.686)	.015 (.005–.022)	.183 (.179–.187)	.745 (.733–.755)
RSF	**.694 (.688–.697)**	Reference	**.181 (.179–.184)**	**.750 (.739–.756)**

Medians of the C-index, C-index differences calculated as (RSF C-index − comparator model C-index), integrated Brier score (IBS), and cumulative/dynamic mean AUC (mean AUC), along with their 95% confidence intervals (CIs), were estimated based on 87 successful iterations out of 200 bootstrap runs. Higher C-index and C/D AUC values indicate better performance, whereas lower IBS values indicate better performance. The best-performing values for each metric are highlighted in bold.

[a] Univariate Cox model.
