# Supplementary material for: Early Prediction of Diabetic Macular Edema via Machine Learning Survival Analysis on Checkup Data
Source: Ophthalmol Sci. 2026 Jun 1;6(8):101262. doi: 10.1016/j.xops.2026.101262 (PMC13355755; doi:10.1016/j.xops.2026.101262)
Supplement: Table S5 [file mmc5.pdf]

Table S5. Sensitivity analysis using different imputation methods

Metrics	Missing imputed by multivariate imputation by chained equations (MICE)	Missing imputed by parameter-specific sample-and-hold approach
C-index	.697 (.672–.719)	.697 (.674–.719)
IBS	.178 (.164–.193)	.180 (.167–.193)
Mean AUC	.740 (.712–.765)	.740 (.714–.764)

Medians (95% bootstraped confidence interval) of the C-index, integrated Brier score (IBS), and cumulative/dynamic mean AUC (mean AUC) are shown.
